# Being Cynical Is Bad for Your Wellbeing: A Structural Equation Model of the Relationship Between Cynicism and Mental Health in First Responders in South Africa

**DOI:** 10.3390/ijerph21121684

**Published:** 2024-12-17

**Authors:** Anita Padmanabhanunni, Tyrone B. Pretorius

**Affiliations:** Department of Psychology, University of the Western Cape, Cape Town 7535, South Africa; apadmana@uwc.ac.za

**Keywords:** anxiety, cynicism, depression, fatigue, first responders, paramedics, police

## Abstract

Cynicism has been associated with adverse mental and physical health outcomes. This study investigated the role of cynicism in relation to indices of mental health among South African first responders. Research has highlighted that first responders are at increased risk of adverse mental health outcomes owing to the nature of their work. The sample consisted of 429 participants who completed a brief demographic questionnaire and several research instruments: Turner Cynicism Scale, Chalder Fatigue Scale, Patient Health Questionnaire-9, and Generalized Anxiety Disorder Scale-7. Structural equation modeling was used to explore the relationship between cynicism and mental health indicators. It included a measurement model, which identified four latent variables—cynicism, fatigue, depression, and anxiety—and their respective indicators, and a structural model, which defined the relationships among these latent variables. Path analysis was used to explore the potential mediating role of fatigue in the relationship between cynicism and indices of mental health. The results demonstrated significant associations between cynicism and anxiety, depression, and fatigue. Fatigue also mediated the relationship between cynicism and indices of mental health. The results underscore the importance of recognizing and addressing cynicism as a critical factor in the mental health of individuals working in high-stress occupations.

## 1. Introduction

Cynicism has been consistently associated with adverse mental and physical health outcomes [1,2]. The construct has been conceptualized as the cognitive component of hostility and is characterized by negative appraisals of the motives of other people, skepticism about the sincerity or integrity of individuals and institutions, and a pessimistic outlook on the likelihood of positive outcomes [3]. While some degree of skepticism can be healthy, excessive cynicism can have detrimental effects on mental health and wellbeing. Cynicism is also recognized as a component of the experience of burnout, particularly in work-related settings. Burnout comprises three dimensions, namely, emotional exhaustion, depersonalization, and personal accomplishment [4]. Cynicism has been conceptualized as part of depersonalization, which manifests as emotional detachment from one’s work and a sense of disillusionment [5]. Individuals experiencing depersonalization often feel disconnected from the people they serve and view their work-related tasks as unimportant or futile. Depersonalization is tied to the stress and exhaustion that accompany prolonged exposure to demanding work environments and is associated with the tendency to disengage or avoid stressful work-related situations [6]. In contrast, cynicism arises as a defensive reaction to the depletion of psychological resources and is related to a reduced likelihood of seeking out emotional or instrumental sources of support [5].

Individuals with greater levels of cynicism tend to experience difficulty generating and implementing adaptive strategies to manage stressors, and this decreased cognitive flexibility is associated with hopelessness, depression, and suicidality [7].

Existing research has also focused on organizational cynicism, which refers to the negative attitudes and beliefs employees hold toward their organization. Organizational cynicism is marked by a belief that the organization lacks integrity, is driven by self-interest, and fails to act in alignment with its stated values or promises [8]. Employees with high levels of organizational cynicism are likely to distrust management, question the organization’s motives, and feel that their contributions are undervalued or unappreciated. This form of cynicism can lead to decreased job satisfaction, reduced commitment, and higher turnover intentions, as employees become disengaged and disillusioned with the organization as a whole [9,10]. Organizational cynicism has been associated with emotional exhaustion, workplace alienation, and reduced work performance [11,12].

The vast majority of studies on cynicism have confirmed that it is negatively related to health outcomes. Cynicism has been associated with cardiovascular disease, diabetes, dementia, and increased mortality [1,13,14]. Individuals with high levels of cynicism have been found to experience increased stress, social isolation, hopelessness, and a reduced likelihood of using mental health care services [2,7,15]. Furthermore, cynicism has also been associated with depression and suicidal ideation [14,16].

Existing studies have emphasized the impact of cynicism on psychological wellbeing across various occupational groups, exploring its role as a mediator and identifying the factors that contribute to its development. For example, a study of medical students reported that increased cynicism was associated with lower psychological wellbeing [2] while a study of nursing staff found that cynicism was associated with vaccination hesitancy [17]. In a study of student teachers, Bakioğlu and Kiraz [18] reported that cynicism was a mediator of the relationship between burnout and wellbeing. Among police officers, cynicism has been highly correlated with job demands and lack of resources [19]. Cynicism was also found to partially mediate the relationship between job stress and a sense of alienation among employees in a hospital setting [20]. Among post-menopausal women, cynicism has been associated with diabetes-related health disparities and the worsening of metabolic syndrome [21].

The current study investigated the role of cynicism in mental health outcomes among a sample of South African first responders. First responders typically include professionals trained specifically to respond to critical events and emergency situations and include police officers, emergency services personnel, and firefighters. Few South African studies have investigated the mental health of first responders. The limited available evidence has highlighted that first responders are at increased risk of exposure to potentially traumatic events in the course of their work [22,23]. This is due the high prevalence of gender-based violence, hijacking, violent crime and road traffic accidents in the country [24,25,26]. In response, some may develop a cynical worldview, which can serve to desensitize them to the emotional impact of their experiences and facilitate their ability to maintain functioning in highly stressful and demanding work environments. However, cynical beliefs have been implicated in precipitating hopelessness, suicidal ideation, and substance use among high risk populations [27]. It can also deter help-seeking behavior and hinder the ability to form supportive relationships. Hence, investigating the role of cynicism among South African first responders can provide important insights into how this coping mechanism may adversely impact health and wellbeing.

International studies have underscored that first responders are at increased risk of adverse mental health outcomes owing to the nature of their work and the COVID-19 pandemic aggravated this susceptibility [28,29]. Emergency service personnel were exposed to additional stressors including fear for their own safety and that of their families, prolonged periods of separation from sources of support, and increased exposure to illness and death. The added stress of working in an environment of uncertainty, with limited resources and heightened personal safety concerns, further exacerbated the mental health burden on these professionals. Studies during and after the pandemic reported rising levels of anxiety, depression, burnout, and post-traumatic stress disorder (PTSD) among first responders. In their systematic review of the literature, Huang and colleagues found that depression, anxiety, and stress were prevalent among first responders for medical emergencies during the COVID-19 pandemic [28]. In a study involving emergency medical service personnel, Vujanovic and colleagues found that first responders exposed to COVID-19 reported increased alcohol use. In addition, worries about vulnerability to infection and the implications of contracting the virus were associated with heightened symptoms of anxiety, depression, and PTSD [29]. Studies have also documented that moral injury has been a significant source of distress among first responders and arises from having to make decisions that violate moral or ethical values [30,31]. This can include situations such as being unable to provide adequate care due to resource limitations, prioritizing certain lives over others during emergencies, or following orders that may conflict with their own sense of right and wrong. For example, during the COVID-19 pandemic, first responders may have faced the painful decision of rationing care or delaying treatment due to overwhelmed healthcare systems. These morally distressing situations can lead to deep emotional and psychological turmoil and aggravate distress [30,32].

Policing has been identified as a mentally and emotionally taxing profession. Law enforcement officers are frequently required to navigate threats to their personal safety, often placing them in dangerous and high-pressure situations. In addition to these physical risks, they face the ongoing stress of having to maintain a constant state of vigilance which can lead to chronic stress and fatigue. Moreover, officers often contend with a perceived or real lack of support from the communities they serve, which can further strain their mental and emotional wellbeing [33,34]. A systematic review and meta-analysis of the prevalence of mental health problems among police officers in 24 countries concluded that depression, PTSD, alcohol dependence, and generalized anxiety disorder were pervasive within this population [34]. In their systematic review, Purba and colleagues concluded that organizational stressors (e.g., long working hours, organizational culture, lack of support, and high workload) were negatively associated with mental wellbeing among police officers [35].

Research on first responder populations in African countries is limited. However, the available evidence underscores significant mental health challenges faced by these professionals. A study of emergency responders in Ethiopia highlighted a high prevalence of PTSD. Prolonged exposure to emergency situations and length of service were among the salient predictors of PTSD [36]. A study of Nigerian firefighters reported high levels of PTSD and highlighted the protective role of resilience and locus of control in reducing vulnerability to post-traumatic stress symptomology [36]. Barnett and colleagues documented the types of work-related traumatic events experienced by police officers in Ghana, identifying road traffic accidents as the most frequent incidents. Their study also highlighted the prevalence of PTSD symptoms within this population [37].

Existing studies have highlighted mental health concerns among South African first responders and factors contributing to wellbeing. In their study of 52 first responders in South Africa, O’Neil and Kruger found that mindset—a combination of dispositional attitudes and coping strategies that help individuals to navigate adversity—was significantly related to perceived wellness [38]. A cross-sectional study of ambulance personnel in a South African province identified chronic organizational stress as one of the significant risk factors for PTSD [22]. Ward and colleagues reported high rates of PTSD and problematic alcohol use among emergency service personnel (e.g., ambulance workers and firefighters) in the country [39]. Fjeldheim and colleagues studied trauma exposure and mental health outcomes among paramedic trainees, finding significant levels of depression, alcohol abuse, chronic perceived stress, and PTSD among the sample [23].

While much research has focused on the mental health of first responders, cynicism as a distinct emotional and cognitive response has received comparatively less attention. Cynicism can affect job performance, decision making, and interpersonal relationships within high-stress professions. Cynicism might also serve as a precursor or correlate to other mental health conditions including PTSD, depression, anxiety, and substance use [12,20,40,41]. Thus, a study focusing on cynicism among first responders could contribute to addressing an important gap in the research on first responder mental health.

The current study aims to contribute to the literature base in this area by using structural equation modeling (SEM) to investigate the role of cynicism as a dispositional factor in mental health among police officers and paramedics in Western Cape Province. The SEM consisted of both a measurement model (the latent variables and their indicators) and a structural model (the relationship between the latent variables).

The study is grounded in the job demands–resources (JD-R) model, which proposes that the balance between job demands (e.g., exposure to trauma) and job resources (e.g., social support and organizational resources) determines employees’ wellbeing. When job demands exceed available resources, it leads to stress and negative emotional outcomes, such as burnout and cynicism [42]. In this context, cynicism may develop as a way for first responders to psychologically detach from overwhelming job demands, allowing them to continue performing their duties [12]. However, the chronic presence of cynicism can erode both job satisfaction and mental health over time.

Given the consistent link between cynicism and adverse mental health outcomes as indicated in the above literature review, we postulated the following hypotheses:

**H1:**  *In the structural model of the SEM the latent variable cynicism would be positively related to the latent variable depression.*

**H2:**  *In the structural model of the SEM the latent variable cynicism would be positively related to the latent variable anxiety.*

**H3:**  *In the structural model of the SEM the latent variable cynicism would be positively related to the latent variable fatigue.*

The literature also reported a link between cynicism and emotional exhaustion [43,44,45] as well as between emotional exhaustion and physical and emotional fatigue [46]. We speculated that fatigue might be the mechanism through which cynicism affects mental health. We, thus, also examined the following hypothesis:

**H4:**  *Fatigue would mediate the relationship between cynicism and indices of mental health.*

## 2. Materials and Methods

### 2.1. Participants

First responders currently employed in Western Cape Province were invited to participate. The sample included 429 first responders, consisting of 309 police officers and 120 paramedics. The study did not impose any exclusion criteria. To capture a diverse range of experiences, participants were recruited from various police stations and healthcare facilities located in both urban and peri-urban regions. We used Google Forms to construct an online version of the questionnaires described in the Instruments section of this paper. Permission was requested from the administrators of Facebook groups consisting of first responders to post the link on their sites together with an invitation to participate in the study. In addition, research assistants visited police stations and hospitals to recruit additional participants. Data collection took place from June 2023 to August 2024.

A description of the sample is presented in Table 1.

Table 1 shows that the majority of the sample were men (56%), were married (51.5%), and worked in an urban setting (92.3%). The mean age of the sample was 39 years (*SD* = 9.9), and the mean length of service was 13.2 years (*SD* = 9.7).

### 2.2. Instruments

Participants completed a brief demographic questionnaire and the following research questionnaires: Turner Cynicism Scale [3], Chalder Fatigue Scale (CFQ-11) [47], Patient Health Questionnaire-9 (PHQ-9) [48], and Generalized Anxiety Disorder Scale (GAD-7) [49].

The Cynicism Scale is an 11-item measure of cynicism or general distrust of the motives of others. Participants respond to the 11 items using a 7-point scale with anchors strongly disagree (1) to strongly agree (7). An example of an item of the Cynicism Scale is “When someone does me a favor, I know they will expect one in return”. In the initial development study of the Cynicism Scale, the authors reported a reliability coefficient of 0.86 and further provided evidence of construct and discriminant validity. We could not find a previous use of the Cynicism Scale in South Africa.

The CFQ-11 is an 11-item measure of the extent and severity of fatigue that includes both physical and psychological fatigue. The CFQ-11 is scored on a 4-point scale ranging from less than usual (0) to much more than usual (3). An example of an item of the CFQ-11 is “Do you have problems with tiredness?” The authors of the CFQ-11 reported a Cronbach’s alpha of 0.89 and provided evidence of face and discriminant validity. A South African study with adolescents living with HIV reported a reliability coefficient of 0.83 and provided evidence of content and construct validity [50].

The PHQ-9 consists of nine items and is used to screen for and measure the severity of depression. Responses to the nine items are given on a 4-point scale that ranges from not at all (0) to nearly every day (3). An example of an item of the PHQ-9 is “Over the last two weeks, how often have you been bothered by feeling tired or having little energy?” In the initial validation study, the authors of the PHQ-9 reported reliability coefficients of 0.89 and 0.86 for two different samples. In addition, the validation study also provided evidence of construct and criterion validity [48]. The PHQ-9 has been extensively used on the African continent, for example, in South Africa, Kenya, and Ghana [51], Tanzania [52], and Nigeria [53], displaying psychometric properties similar to those in the original validation study.

The GAD-7 consists of seven items and is used to screen for and measure the severity of generalized anxiety disorder. The GAD-7 is scored on a 4-point scale that ranges from not at all (0) to nearly every day (3). An example of an item from the GAD-7 is “Over the last two weeks, how often have you been bothered by feeling nervous, anxious, or on edge?” The authors of the scale reported a reliability coefficient of 0.92 and also provided evidence for procedural, construct, criterion, and factorial validity [49]. The GAD-7 is commonly used in South Africa to assess levels of anxiety, and reported reliabilities are similar to the original validation study: for example, a study of tuberculosis patients reported a reliability coefficient of 0.86 [54]; with university students, a reliability of 0.89 was reported [55]; and with a large sample of adult workers (*n* = 1816), a reliability of 0.89 was reported [56].

### 2.3. Ethics

The research was conducted according to the guidelines of the Declaration of Helsinki and received ethical clearance from the Humanities and Social Sciences Research Ethics Committee of the University of the Western Cape (ethics reference: HS23/2/4, 23 May 2023) and the South African Police Services (ethics reference: 3/34/2, 27 June 2023) and approval letters from the Western Cape Department of Health (to access hospitals, reference: WC_202307_041, 15 September 2023; and from a private ambulance company to approach their employees, reference: 12 December 2023). Participation was voluntary, participants provided informed consent on the first page of the electronic questionnaire, and no personal information was collected.

### 2.4. Data Analyses

IBM SPSS for Windows, version 29 (IBM Corp., Armonk, NY, USA) was used to determine descriptive statistics (means and standard deviations), the intercorrelations between variables (Pearson *r*), and the estimates of internal consistency (Cronbach’s alpha and McDonald’s omega) for the scales. Before any formal analysis, we examined the distribution of data for the scales by using indices of skewness and excess kurtosis. Skewness values between −2 and +2 [57] and excess kurtosis between −1 and +1 [58] would be indicative of a normal distribution.

IBM Amos for Windows, version 28 (IBM Corp., Armonk, NY, USA) was used to assess the relationship between cynicism and the indices of mental health using a structural equation model. The structural equation model consisted of both a measurement model, which reflected the four latent variables and their respective indicators, and a structural model, which defined the relationship between the four latent variables. Model fit was examined with the following recommended fit indices [59]:

- Chi-squared (χ^2^), which should be non-significant; however, this would indicate a perfect fit [60].
- Relative chi-squared (χ^2^/*df*); a value ≤3 indicates good fit.
- Comparative fit index (CFI), which should be ≥0.90 [59].
- Root mean square error of approximation (RMSEA), which should be ≤0.08.
- Standardized root mean square residual (SRMR), which should be ≤0.08.

In the measurement model, factor loadings should be significant and greater than 0.50, indicating that all the items contribute to the measurement of the latent variable [61]. In the structural model, the correlations between the latent variables should be significant.

To examine the mediating role of fatigue we used path analysis with cynicism as the independent variable, depression and anxiety as dependent variables, and fatigue as the mediator variable. The significance of the mediating effects was assessed using bootstrapped 95% confidence intervals.

## 3. Results

The zero-order correlations between variables, descriptive statistics, and reliability coefficients are reported in Table 2.

Table 2 indicates that the skewness values, which ranged between −0.62 and 0.38, fell in the recommended range of −2 to +2. Similarly, the kurtosis values ranged between −0.81 and 0.84 and were thus also within the recommended range of −1 to +1. These skewness and kurtosis values would therefore indicate that the data for all the variables were approximately normally distributed. Table 2 further shows that cynicism was significantly related to depression (*r* = 0.21, *p* < 0.001), anxiety (*r* = 0.22, *p* < 0.001), and fatigue (*r* = 0.21, *p* < 0.001). The positive relationships indicate that higher levels of cynicism are associated with higher levels of depression, anxiety, and fatigue. Lastly, Table 2 shows that all of the scales had satisfactory reliability (α and ω = 0.87 to 0.92).

**Figure 1:**
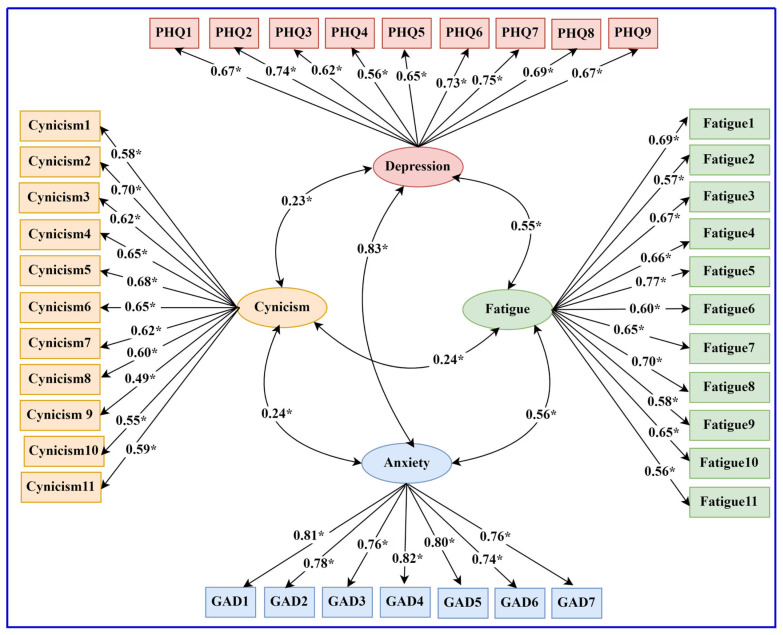
Structural equation model of the relationship between cynicism and indices of mental health. Note: Rectangles indicate observed variables; ellipses indicate latent variables. All regression coefficients are standardized; * *p* < 0.001.

The structural equation model showing the latent variables and their indicators (measurement model) and the relationship between cynicism, depression, anxiety, and fatigue is shown in Figure 1.

The fit indices for the structural equation model (χ^2^ = 1154.57, *p* < 0.001, relative χ^2^ = 1.83, CFI = 0.94, SRMR = 0.05, and RMSEA = 0.04) all met the thresholds for acceptable model fit. In the measurement model, all the factor loadings for the four scales were significant. For the Cynicism Scale, only one item had a loading slightly below 0.50 (i.e., 0.49); all other loadings ranged between 0.55 and 0.70. In the case of the PHQ, the loadings ranged between 0.56 and 0.75; for the GAD, between 0.74 and 0.82; and for the Fatigue Scale, between 0.56 and 0.77. The structural model indicated that cynicism was significantly related to the indices of mental health, thus confirming all the hypotheses regarding the relationship between cynicism and indices of mental health.

The direct and indirect effects of cynicism on depression and anxiety as obtained in the mediation analysis are presented in Table 3.

**Figure 2:**
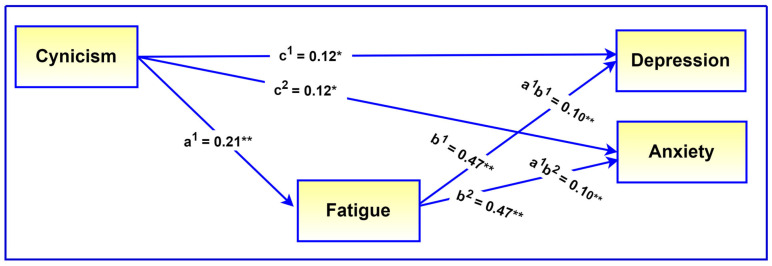
Illustration of the mediating role of fatigue. Note: All regression coefficients are standardized. c^1^–c^2^ = direct effects of predictor on dependent variables, a^1^ = direct effect of predictor on mediator, b^1^–b^2^ = direct effects of mediator on dependent variables, a^1^b^1^, a^1^b^2^, mediating effects. * *p* < 0.01, ** *p* < 0.001.

Table 3 indicates that the indirect effects of cynicism on depression (β = 0.10, 95% CI = [0.04, 0.09], *p* < 0.001) and anxiety (β = 0.10, 95% CI = [0.04, 0.08], *p* < 0.001) were significant, confirming a mediating role for fatigue. Since the direct effects of cynicism on depression (β = 0.12, 95% CI = [0.02, 0.13], *p* = 0.005) and anxiety (β = 0.12, 95% CI = [0.02, 0.12], *p* = 0.004) were also significant in the presence of the mediator, this would be indicative of partial mediation. The mediating role of fatigue is visually presented in Figure 2.

## 4. Discussion

The current study investigated the role of cynicism in mental health outcomes among South African first responders. There were several salient findings. First, the study found that cynicism was significantly related to internalizing disorders, specifically anxiety and depression. This suggests that first responders who exhibit higher levels of cynicism are more likely to experience symptoms associated with anxiety and depressive disorders. The relationship between cynicism and internalizing disorders may be attributed to the emotional detachment and negative outlook associated with cynicism, which can exacerbate feelings of hopelessness, sadness, and worry. Sociocognitive theories propose that an individual’s cognitive appraisals of stressful events is central in determining their emotional and behavioral responses [62,63]. These theories suggest that the way individuals interpret and evaluate adverse life events significantly affects their mental health outcomes. In the context of cynicism, a cynical outlook may skew these appraisals toward negative interpretations, where first responders may view challenges and adversities as more threatening or insurmountable than they actually are. This negative cognitive appraisal can increase the likelihood of experiencing anxiety and depression, as the individual feels overwhelmed by stressors that are perceived as pervasive and unmanageable. Furthermore, cynicism has been associated with reduced cognitive flexibility and a lower likelihood of accessing sources of support, which can perpetuate psychological distress [7]. The findings highlight the importance of addressing cynicism within this population to mitigate its impact on mental health. Existing studies have suggested that developing psychological resources such as self-efficacy, hope, and resilience can serve to mitigate the likelihood of cynicism among employees [64,65]. Cognitive-behavioral interventions have been efficacious in promoting these psychological resources by identifying and challenging problematic thinking patterns, enhancing self-awareness, promoting adaptive problem solving, and improving emotional regulation capacities [66]. These types of intervention could be implemented as part of employee wellness initiatives and integrated into routine organizational practices.

Second, we found that cynicism was significantly associated with fatigue. This association indicates that individuals who exhibit higher levels of cynicism are more likely to experience feelings of tiredness, exhaustion, and lack of energy. Cynicism, characterized by a negative and distrustful outlook, can lead to emotional and mental strain, which, over time, can contribute to physical fatigue. This fatigue might result from the chronic stress and emotional detachment that cynicism fosters, making it difficult for first responders to find meaning or satisfaction in their work. The link between cynicism and fatigue also suggests that cynical attitudes may impair one’s ability to recover from work-related stressors effectively, leading to prolonged periods of tiredness. This can affect a first responder’s performance, decision-making abilities, and overall health, further contributing to a cycle of stress and exhaustion. Existing interventions focusing on addressing fatigue have highlighted the essential role of organizational factors including work hours, workload management, and support systems. Managing work hours to ensure adequate rest and recovery time can significantly reduce physical and mental exhaustion. Effective workload management entails balancing tasks to prevent overload and ensuring employees have manageable schedules [67]. Providing access to mental health professionals, peer support groups, and stress management programs can further support first responders in managing their mental health effectively. Cognitive-behavioral interventions and graded exercise therapy have been found to be effective in reducing fatigue among various populations and could form part of interventions to promote adaptation and coping among first responders [68,69].

The JD-R model posits that job demands, such as exposure to trauma and chronic stress, can lead to negative outcomes when adequate resources are lacking [42]. In this study, cynicism emerged as a crucial variable that can potentially play a role in the impact of these job demands on mental health issues, such as anxiety and depression. Cynicism, often arising as a response to overwhelming job demands, can exacerbate emotional detachment and negative cognitive appraisals, which in turn increase the risk of internalizing disorders. Furthermore, the association between cynicism and fatigue highlights the role of emotional strain in depleting psychological and physical resources, making it harder for first responders to recover from stressors. These findings lend support for the JD-R model and underscore the need for interventions that strengthen internal resources and capacities (e.g., resilience and emotional regulation skills).

Thirdly, we found that fatigue is the pathway through which cynicism impacts mental health. In this regard fatigue was found to partially mediate the relationship between cynicism and depression as well as anxiety. This would indicate that excessive cynicism is associated with higher levels of fatigue which in turn is associated with higher levels of depression and anxiety. While studies directly examining fatigue as a mediator are limited, existing research on emotional exhaustion—a construct closely related to fatigue—has consistently highlighted its mediating role in mental health outcomes. For instance, Zhang and colleagues reported that emotional exhaustion mediated the relationship between work–family conflict and anxiety among female medical staff [70]. Similarly, Xu and colleagues found that emotional exhaustion mediated the relationship between sleep disturbances and depressive symptoms among nurses [71]. Furthermore, Santa Maria and colleagues, in a study of police officers, reported that emotional exhaustion mediated the relationship between job demands and depression and anxiety [72].

The study has several limitations. First, the study is cross-sectional in nature, and while the findings indicate associations, they do not establish a cause-and-effect relationship. Longitudinal studies are needed to explore how cynicism develops over time and its long-term impact on mental health. This could provide insights into whether cynicism is a stable trait or whether it fluctuates in response to specific job demands or changes in organizational resources. Second, the reliance on self-reported measures for assessing cynicism and mental health outcomes may introduce bias, as participants might underreport or overreport their symptoms due to social desirability or lack of self-awareness. Qualitative studies of first responders may provide an important avenue to fully understanding the lived experience of cynicism and its impact on mental health among first responders. Third, the study focused on first responders in South Africa, which may limit the generalizability of the findings to other populations or geographic regions. It would be useful for future studies to compare these findings with those in other regions to determine the role of cultural, organizational, and societal factors in cynicism.

## 5. Conclusions

To the authors’ knowledge, this is the first South African study to highlight the significant role of cynicism in influencing mental health outcomes among first responders. The findings indicate that cynicism is associated with higher levels of internalizing disorders, such as anxiety and depression, and is linked to increased fatigue. These results underscore the importance of recognizing and addressing cynicism as a critical factor in the mental health of individuals working in high-stress occupations. Addressing cynicism through targeted interventions and organizational support is essential for improving the psychological wellbeing of first responders.

## Figures and Tables

**Table 1:** Demographic and Work-Related Characteristics of Participants.

Variable	Category	*n*	%	Mean	*SD*
Gender	Male	236	56%		
	Female	193	45%		
Relationship status	Single	151	35.2%		
	Married	221	51.5%		
	Divorced/Separated/Widowed	57	13.3%		
Work setting	Urban	396	92.3%		
	Peri-urban	11	2.6%		
	Rural	22	5.1%		
Age				39.0	9.9
Length of service				13.2	9.7

*SD* = Standard Deviation.

**Table 2:** Intercorrelations, descriptive statistics, and reliabilities.

Variable/Scale	1	2	3	4
1. Cynicism	—			
2. Depression	0.21 **	—		
3. Anxiety	0.22 **	0.73 **	—	
4. Fatigue	0.21 **	0.49 **	0.50 **	—
Mean	52.46	9.51	7.73	14.40
*SD*	10.16	6.42	5.86	6.80
Skewness	−0.62	0.37	0.38	0.12
Kurtosis	0.84	−0.49	−0.81	−0.25
α	0.88	0.89	0.92	0.89
ω	0.87	0.89	0.92	0.89

** *p* < 0.001; α = Cronbach’s alpha; ω = McDonald’s omega.

**Table 3:** Results of mediation analysis.

Effects	B	SE	95% CI	β	*p*
Direct effects					
Cynicism → Depression	0.08	0.03	[0.02, 0.13]	0.12	0.005
Cynicism → Anxiety	0.07	0.03	[0.02, 0.12]	0.12	0.004
Fatigue → Depression	0.44	0.04	[0.37, 0.51]	0.47	<0.001
Fatigue → Anxiety	0.41	0.04	[0.34, 0.47]	0.47	<0.001
Indirect effects					
Cynicism → Fatigue → Depression	0.06	0.02	[0.04, 0.09]	0.10	<0.001
Cynicism → Fatigue → Depression	0.06	0.02	[0.04, 0.08]	0.10	<0.001

B = unstandardized coefficient; SE = standard error; CI = confidence interval; β = standardized coefficient; *p* = significance level.

## Data Availability

The original data presented in the study are openly available in FigShare at https://doi.org/10.25379/uwc.26862640.v1 (accessed on 1 September 2024).
